# Association of pelvic inflammatory disease (PID) with ectopic pregnancy and preterm labor in Taiwan: A nationwide population-based retrospective cohort study

**DOI:** 10.1371/journal.pone.0219351

**Published:** 2019-08-13

**Authors:** Chun-Chung Huang, Chien-Chu Huang, Shao-Yi Lin, Cherry Yin-Yi Chang, Wu-Chou Lin, Chi-Hsiang Chung, Fu-Huang Lin, Chang-Huei Tsao, Chun-Min Lo, Wu-Chien Chien

**Affiliations:** 1 Department of Biomedical Engineering, National Yang-Ming University, Taipei, Taiwan; 2 Department of Obstetrics and Gynecology, China Medical University Hospital, Taichung, Taiwan; 3 Graduate Institution of Biomedical Sciences, China Medical University, Taichung, Taiwan; 4 Department of Mechanical and Computer-Aided Engineering, National Formosa University, Yunlin, Taiwan; 5 China Medical University, Department of Medicine, Taichung, Taiwan; 6 School of Public Health, National Defense Medical Center, Taipei, Taiwan; 7 Department of Medical Research, Tri-Service General Hospital, Taipei, Taiwan; 8 Department of Microbiology & Immunology, National Defense Medical Center, Taipei, Taiwan; 9 Graduate Institution of Life Science, National Defense Medical Center, Taipei, Taiwan; 10 School of Public Health, National Defense Medical Center, Taipei, Taiwan; University of North Carolina at Chapel Hill, UNITED STATES

## Abstract

**Background:**

Pelvic inflammatory disease (PID) is an infectious disease that causes tubal occlusion and other pelvic and abdominal adhesions. The incidence of pelvic inflammatory disease (PID) has increased due to the sexually active status of the young population. This leads to a more serious problem and a larger effect than previously observed. However, there have been few studies on this topic in Asian populations.

**Aim:**

We aimed to evaluate the risk of preterm labor and/or ectopic pregnancy in Taiwanese women following PID.

**Design:**

Using the Taiwan National Health Insurance Database, we designed a retrospective cohort study that included 12- to 55-year-old pregnant women between 2000 and 2010. We selected a 1:3 age-matched control group of non-PID women. The endpoint was any episode of preterm labor or ectopic pregnancy; otherwise, the patients were tracked until 31 December 2010.

**Methods:**

The risk factors for preterm labor or ectopic pregnancy were explored. For cases included from the index date until the end of 2010, we analyzed the risk of incident preterm labor or ectopic pregnancy. With the use of a multivariate Cox proportional hazard regression analysis, we calculated the hazard ratio (HR) with a 95% CI and compared it with that of the control group.

**Results:**

This study examined 30,450 patients with PID and 91,350 controls. During the follow-up period, patients in the PID group were more likely to develop preterm labor or ectopic pregnancy than patients in the control group. The cumulative incidence rates for developing preterm labor were 1.84% (561/30,450 individuals) in patients with PID and 1.63% (1492/91,350 individuals) in patients without PID. On the other hand, the cumulative incidence rate for developing ectopic pregnancy in patients with PID was 0.05% (14/30,450 individuals) but was only 0.04% (33/91,350 individuals) in patients without PID. Compared with those without PID, the patients with PID had a 1.864 times (P<0.001) higher risk of developing preterm labor and a 2.121 times (P = 0.003) higher risk of developing ectopic pregnancy.

**Conclusion:**

Our study provided evidence of an increased risk of preterm labor or ectopic pregnancy in PID patients.

## Background

Pelvic inflammatory disease (PID) is an infectious and inflammatory disease of the upper female genital tract, including the uterus, fallopian tubes, and related pelvic organs. PID is a polymicrobial infection typically observed in sexually active females. When microorganisms ascend from the lower genital tract into the upper genital tract, PID gradually develops. The clinical presentation of PID varies in severity, with most patients presenting with mild disease[1]. The diagnosis is sometimes difficult to establish; practical diagnostic methods include a careful history and physical examination (including pelvic examination), laboratory tests (including blood samples and, particularly, a cervical Gram stain or cervical culture result), and sometimes culdocentesis[2–4]. Many women experience a clinically silent spread of infection to the upper genital tract, which results in subclinical PID [5].

In a famous multicenter, randomized clinical trial designed for PID in North America, the PEACH trial, upper genital tract detection of gonorrhea, chlamydia, or endometritis was sufficient to confirm a diagnosis of PID[6]. The PEACH trial results showed that there were no differences in reproductive health outcomes between women with and without endometritis or with upper genital tract infection[7]. However, other studies revealed that a history of pelvic inflammatory disease prior to admission was associated with infertility, preterm labor, chronic pelvic pain and ectopic pregnancy[8–12].

Both obstetricians and gynecologists focus on women's health, and the prevention of pregnancy complications is the main concern. Ectopic pregnancy is a complication of early pregnancy, and preterm labor is a complication that occurs in the second and third trimesters. Getting pregnant and delivering a healthy baby is an important issue for women, families and society. Preterm labor and ectopic pregnancy can have negative consequences on obstetric results and on a woman’s psychological health. Preterm labor and ectopic pregnancy are two independent events, and they have different pathophysiologies. However, both of these conditions share the same risk factor: infection or a previous infectious episode.

Tubal occlusion was found to be diagnosed in 12.8% of patients after one infection, in 35.5% of patients after two infections, and in 75% of patients after three or more infections [13]. Some studies have revealed that the tubal adhesion caused by PID may increase the possibility of ectopic pregnancy [14–19]. At the same time, studies with small sample sizes and single hospital studies have found that both upper and lower genital tract infections, such as PID [20,21] and bacterial vaginosis, are increasingly associated with adverse consequences in obstetrics, such as preterm membrane rupture, preterm labor and preterm birth [20–31]. PID and amnionitis may result in poor outcomes of subsequent pregnancies[32]. However, the effect of PID on ectopic pregnancy and preterm labor has not been studied in recent years or in Taiwanese or Asian populations.

Therefore, we conducted a population-based study utilizing data from a nationwide health insurance database, the Taiwan National Health Insurance Research Database (NHIRD), to examine the risk of developing ectopic pregnancy and preterm labor among patients with PID.

## Materials and methods

### Data sources

In this study, we used data from the Taiwan National Health Insurance Research Database (NHIRD) to investigate the risk of ectopic pregnancy or preterm labor in patients with PID over a 10-year period. We reviewed records from 2000 to 2010 in the Health Insurance Database, which constitutes a valid representative sample of the total population in Taiwan. The NHIRD has been documenting the medical information of all insured patients since 1995. The national database includes a population of 23.74 million individuals, and it reached 99.6% coverage in 2009[33]. Each small databank for the study was from one million individuals randomly recruited from the NHIRD. The diagnostic and treatment codes in the NHIRD application forms were based on the International Classification of Diseases, Ninth Revision, and Clinical Modification (ICD-9-CM) during the study period. The use of data for our study was permitted by the National Health Research Institute. This study was approved by the Institutional Review Board of Tri-Service General Hospital (IRB No. 2-105-05-082).

### Study design and sampled participants

This study utilized a retrospective matched-cohort design. Among the 986,713 individuals recorded from the outpatient and inpatient data from January 1, 2000, to December 31, 2010, 32,239 individuals were diagnosed with PID (ICD-9-CM codes 614.9) prior to the index date. Patients were excluded if they met one of the following criteria: were diagnosed with PID before the index date, had preterm labor or ectopic pregnancy before tracking, were aged <12 years or > 55 years, and were male.

**Fig 1:**
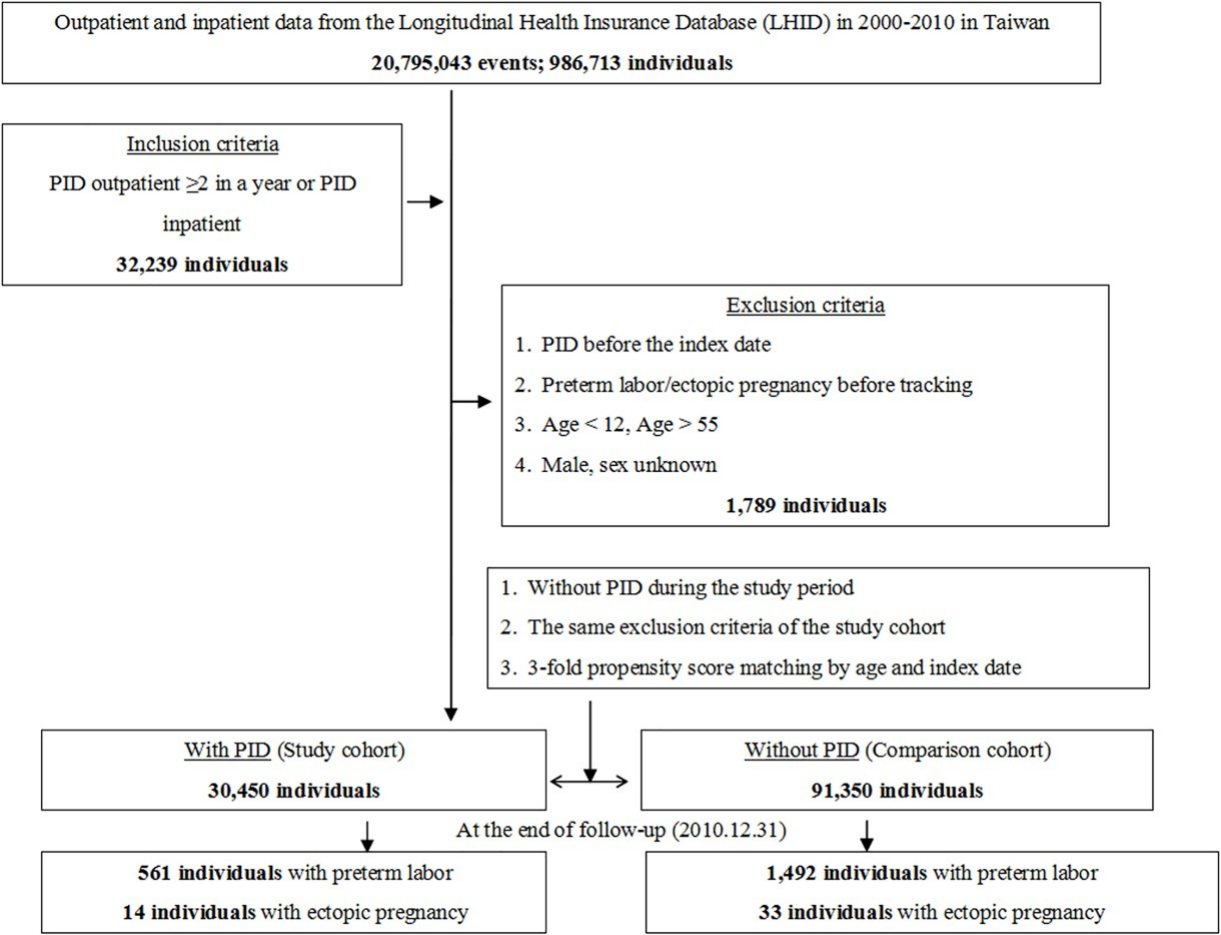
The flowchart of study sample selection from the National Health Insurance Research Database in Taiwan. PID = Pelvic inflammatory disease: ICD-9-CM 614.9. Preterm labor: ICD-9-CM 644. Ectopic pregnancy: ICD-9-CM 633.

Ultimately, the PID group consisted of 30,450 individuals. The control group had the same exclusion criteria as the case group, but individuals in the control group did not have PID during the study period. The controls were matched 3:1 by index date and age, and the control group included 91,350 individuals. The tracking of the case and control groups continued until December 31, 2010. Tracking ended with the occurrence of preterm labor or ectopic pregnancy. Individuals having had at least two diagnoses of PID (ICD-9-CM code 614.9) according to a gynecologist at a minimum of two visits per year were defined as diagnosed with PID (Fig 1).

### Outcome measures

Our study participants were followed from the index date until the onset of preterm labor (ICD-9-CM 644.0–644.9) or ectopic pregnancy (ICD-9-CM 633.0–633.9), until the withdrawal from the National Health Insurance (NHI) program, or until the end of 2010.

### Covariates

The covariates included the age group, geographical area of residence, urbanization level of residence (level 1 to 4), number of pregnancies and monthly income. The age groups were categorized as 12–19, 20–29, 30–39, 40–49, or 50–55 years old. The geographical areas of residence were categorized as northern, central, southern, or eastern Taiwan or outlet islands. The urbanization level of residence was defined according to the population and various indicators of the level of development. Level 1 was defined as a population >1,250,000 people and with a specific designation as political, economic, cultural or metropolitan development. Level 2 was defined as a population between 500,000 and 1249,999 people that played an important role in the political system, economy, and culture. Urbanization levels 3 and 4 were defined as a population between 149,999 and 499,999 and <149,999, respectively. The monthly income was categorized into three groups in New Taiwan Dollars [NTD]: <18,000, 18,000 to 34,999, and >35,000. Baseline comorbidities included diabetes mellitus (ICD-9-CM code 250), hypertension (ICD-9-CM codes 401–405), hyperlipidemia (ICD-9-CM code 272), obesity (ICD-9-CM codes 278), heart disease (ICD-9-CM codes 410–429), and chronic kidney disease (CKD) (ICD-9-CM code274.1, 403–404, 440.1, 442.1, 447.3, 572.4, 580–589, 642.1, 646.2).

### Statistical analyses

All analyses were performed using SPSS 21 software (SPSS, Inc., Chicago, IL, USA). Chi-square and t tests were used to evaluate the distributions of categorical and continuous variables, respectively.

Differences in the distribution of age, insurance premiums, comorbidities, season, location, urbanization level, level of care between the two groups and between subjects with and without ectopic pregnancy or preterm labor were compared using the chi-square test. Multivariate Cox proportional hazard regression analysis was used to determine the risk of preterm labor and ectopic pregnancy, and the results are presented as hazard ratios (HRs) with 95% confidence intervals (CIs). The preterm labor and/or ectopic pregnancy risk difference between the two groups was estimated using the Kaplan-Meier method along with the log-rank test. The results were considered statistically significant if two-tailed p values were less than 0.05.

## Results

**Fig 2:**
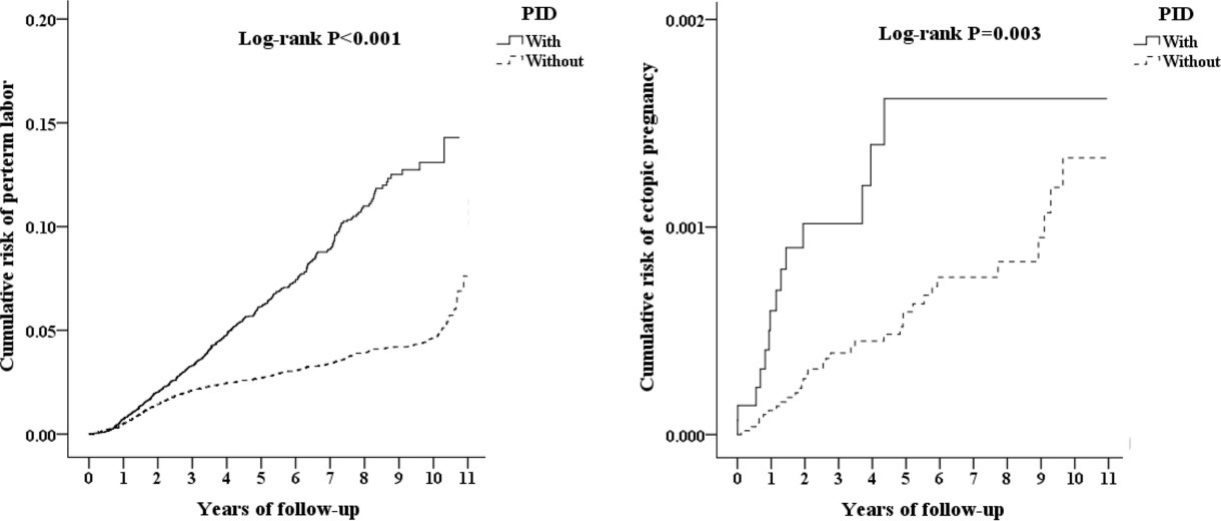
Kaplan-Meier analysis for the cumulative risk of preterm labor/ectopic pregnancy among females aged 12–55 stratified by pelvic inflammatory disease (PID) with the log-rank test. Left-button events: preterm labor. Right-button events: ectopic pregnancy.

This study examined 30,450 patients with PID and 91,350 controls. Table 1 shows the demographic characteristics of the case and control groups at the end of follow-up. At the 10-year follow-up, patients with a previous PID history had a significantly higher risk of developing preterm labor or ectopic pregnancy than patients without PID. The cumulative incidence rates for developing preterm labor were 1.84% (561/30,450 individuals) in patients with PID and 1.63% (1492/91,350 individuals) in patients without PID. On the other hand, the cumulative incidence rate for developing ectopic pregnancy in patients with PID was 0.05% (14/30,450 individuals) but was only 0.04% (33/91,350 individuals) in patients without PID. The Kaplan-Meier analysis indicated that patients with PID had a significantly higher risk of developing preterm labor or ectopic pregnancy than patients without PID (log-rank test p <0.001; p = 0.003) (Fig 2).

**Table 1:** Characteristics of the study endpoints.

PID	Total		With		Without		P
Variables	n	%	n	%	n	%	
**Total**	121,800		30,450	25.00	91,350	75.00	
**Preterm labor**	2,053	1.69	561	1.84	1,492	1.63	0.014
**Ectopic pregnancy**	47	0.04	14	0.05	33	0.04	0.050
**Age group (years)**							<0.001
12–19	4,280	3.51	1,347	4.42	2,933	3.21	
20–29	29,497	24.22	8,194	26.91	21,303	23.32	
30–39	31,841	26.14	9,091	29.86	22,750	24.90	
40–49	40,502	33.25	9,287	30.50	31,215	34.17	
50–55	15,680	12.87	2,531	8.31	13,149	14.39	
**Insurance premium (NT$)**							<0.001
<18,000	118,694	97.45	29,759	97.73	88,935	97.36	
18,000–34,999	2,241	1.84	530	1.74	1,711	1.87	
≥35,000	865	0.71	161	0.53	704	0.77	
**DM**	4,590	3.77	951	3.12	3,639	3.98	<0.001
**HTN**	5,030	4.13	920	3.02	4,110	4.50	<0.001
**Hyperlipidemia**	1,183	0.97	240	0.79	943	1.03	<0.001
**Obesity**	88	0.07	24	0.08	64	0.07	0.629
**Heart disease**	4,694	3.85	1,226	4.03	3,468	3.80	0.073
**CKD**	1,734	1.42	271	0.89	1,463	1.60	<0.001
**Season**							<0.001
Spring	29,400	24.14	7,218	23.70	22,182	24.28	
Summer	31,436	25.81	7,994	26.25	23,442	25.66	
Autumn	32,529	26.71	8,427	27.67	24,102	26.38	
Winter	28,435	23.35	6,811	22.37	21,624	23.67	
**Location**							<0.001
Northern Taiwan	52,242	42.89	13,760	45.19	38,482	42.13	
Central Taiwan	33,199	27.26	7,632	25.06	25,567	27.99	
Southern Taiwan	30,132	24.74	7,345	24.12	22,787	24.94	
Eastern Taiwan	5,768	4.74	1,605	5.27	4,163	4.56	
Outlet islands	459	0.38	108	0.35	351	0.38	
**Urbanization level**							<0.001
1 (The highest)	42,479	34.88	10,287	33.78	32,192	35.24	
2	52,363	42.99	13,308	43.70	39,055	42.75	
3	10,778	8.85	2,728	8.96	8,050	8.81	
4 (The lowest)	16,180	13.28	4,127	13.55	12,053	13.19	
**Level of care**							<0.001
Hospital center	38,369	31.50	10,535	34.60	27,834	30.47	
Regional hospital	43,302	35.55	13,473	44.25	29,829	32.65	
Local hospital	40,129	32.95	6,442	21.16	33,687	36.88	

P-value (category variable: chi-square/Fisher’s exact test; continuous variable: t-test)

* Abbreviations: DM: Diabetes mellitus, HTN: Hypertension, CKD: Chronic kidney disease

Compared to the controls, patients with PID tended to have lower insurance premiums (97.73% V.S. 97.36%; p<0.001) and had lower rates of DM (3.12% V.S. 3.98%; p<0.001), HTN (3.02% V.S. 4.50%; p<0.001), hyperlipidemia (0.79% V.S. 1.03; p<0.001) and CKD (0.89% V.S. 1.60%; p<0.001). Regarding the season of hospital visits, patients with PID had more frequent visits in summer (26.25% V.S. 25.66%; p<0.001) and autumn (27.67% V.S. 26.38%; p<0.001) than controls. Patients with PID lived more often in less urbanized areas and in northern areas (45.19% V.S. 42.13%) and in eastern Taiwan (5.27% V.S. 4.56%) (P<0.001) than controls. Compared to the controls, more patients with PID were treated in the hospital center (34.60% V.S. 30.47%) and in regional hospitals (44.25% V.S. 32.65%) (P<0.001). Regarding the ages between the two groups, there was a significantly higher percentage of patients between the ages of 12 and 39 in the PID group than in the control group (61.19% V.S. 51.43%; p<0.001) (Table 1).

A Cox regression analysis of the factors associated with the risk of preterm labor and ectopic pregnancy was performed. After adjusting for season, urbanization level of residence, location, number of births and monthly income, the patients with PID had a 1.864 times (P<0.001) higher risk of developing preterm labor and a 2.121 times (P = 0.003) higher risk of developing ectopic pregnancy than patients without PID (Table 2 and S1 Table).

**Table 2:** Factors of preterm labor/ectopic pregnancy by using Cox regression.

Events	Preterm labor				Ectopic pregnancy			
Variables	Adjusted HR	95% CI	95% CI	P	Adjusted HR	95% CI	95% CI	P
**PID**								
Without	Reference				Reference			
With	1.864	1.482	2.062	<0.001	2.121	1.803	3.776.	0.003
**Number of births**								
1	Reference				Reference			
≥2	0.993	0.482	1.795	0.513	1.024	0.589	2.131	0.330
**Age group (years)**								
12–19	Reference				Reference			
20–29	0.607	0.462	0.797	<0.001	0.343	0.079	1.484	0.152
30–39	0.297	0.226	0.390	<0.001	0.192	0.044	0.842	0.029
40–49	0.025	0.017	0.036	<0.001	0.083	0.016	0.426	0.003
50–55	0.000	-	-	0.705	0.000	-	-	0.933
**HTN**	1.057	1.008	1.406	0.004	1.496	0.194	11.517	0.699
**Heart disease**	1.229	1.130	1.405	<0.001	1.792	1.108	5.831	<0.001
**CKD**	1.404	1.092	1.849	0.017	2.891	0.378	22.120	0.307
**Level of care**								
Hospital center	1.275	1.069	1.626	0.010	1.883	1.669	2.126	<0.001
Regional hospital	1.194	1.026	2.576	0.038	1.475	1.324	1.644	<0.001
Local hospital	Reference				Reference			

HR = hazard ratio, CI = confidence interval, adjusted HR: adjusted variables listed in the table

Adjusted variables: geographical area of residence, urbanization level of residence, monthly income, season, diabetes mellitus, hyperlipidemia, and obesity

Comparing the different age groups, those aged between 12 and 19 years had a significantly higher risk of developing preterm labor than those aged between 20 and 29 (0.607-fold, P<0.001), 30 and 39 (0.297-fold, P<0.001), and 40 and 49 (0.025-fold, P<0.001). Those aged between 12 and 19 years had a significantly higher risk of developing ectopic pregnancy than those aged between 30 and 39 years (0.192-fold, P = 0.029) and between 40 and 49 (0.083-fold, P = 0.003) years old. Those with hypertension (HTN) (P = 0.004), heart disease (P<0.001), and chronic kidney disease (CKD) (P = 0.017) were associated with a higher risk of developing preterm labor than those without these comorbidities. Additionally, those with heart disease (P<0.001) were associated with a higher risk of developing ectopic pregnancy than those without these comorbidities. A higher incidence of preterm labor or ectopic pregnancy development was observed among PID patients who visited the hospital center and regional hospitals than among those who visited local hospitals (Table 2).

The incidence and HR of preterm labor or ectopic pregnancy in populations with or without PID relative to those of the controls are listed in Table 3.

**Table 3:** Factors of preterm labor/ectopic pregnancy stratified by the variables listed in the table by using Cox regression.

Events	Preterm labor								Ectopic pregnancy							
PID	With PID		Without PID		With PID vs. Without PID *(Reference)*				With PID		Without PID		With PID vs. Without PID *(Reference)*			
Stratified	Events	Rate	Events	Rate	Adjusted HR	95% CI	95% CI	P	Events	Rate	Events	Rate	Adjusted HR	95% CI	95% CI	P
**Total**	561	1,208.18	1,492	532.46	1.864	1.482	2.062	<0.001	14	30.15	33	11.78	2.121	1.803	3.776.	0.003
**Age group (years)**																
12–19	12	3,215.26	43	2,777.33	0.969	0.878	1.069	0.264	0	0	2	129.18	0.000	-	-	0.842
20–29	295	2,801.93	725	1,527.55	1.535	1.391	1.694	<0.001	8	75.98	12	25.28	2.649	1.403	5.007	<0.001
30–39	240	1,543.12	683	788.92	1.637	1.484	1.806	<0.001	5	32.15	14	16.17	1.753	0.928	3.312	<0.001
40–49	14	111.54	41	66.20	1.410	1.278	1.556	<0.001	1	7.97	5	8.07	0.870	0.461	1.644	0.597
50–55	0	0	0	0	-	-	-	-	0	0	0	0	-	-	-	-
**Insurance premium (NT$)**																
<18,000	543	1,200.98	1,458	534.11	1.882	1.705	2.076	<0.001	13	28.75	30	10.99	2.307	1.221	4.359	<0.001
18,000–34,999	15	1,478.78	27	479.19	2.583	2.341	2.849	<0.001	1	98.59	2	35.50	2.449	1.296	4.627	<0.001
≥35,000	3	1,453.28	7	438.85	2.772	2.512	3.058	<0.001	0	0	1	62.69	0.000	-	-	0.994
**DM**																
Without	557	1,280.27	1,480	576.41	1.859	1.685	2.051	<0.001	14	32.18	33	12.85	2.207	1.169	4.171	<0.001
With	4	136.65	12	51.18	2.234	2.025	2.465	<0.001	0	-	0	-	-	-	-	-
**HTN**																
Without	561	1,288.94	1,491	588.09	1.834	1.662	2.024	<0.001	13	29.87	33	13.02	2.023	1.071	3.823	<0.001
With	0	0	1	3.75	0.000	-	-	0.894	1	34.37	0	0.00	∞	-	-	0.987
**Hyperlipidemia**	561	1,227.64	1,492	543.45	1.899	1.721	2.095	<0.001	14	30.64	33	12.02	2.257	1.195	4.265	0.012
**Obesity**	561	1,209.77	1,492	533.14	1.899	1.721	2.095	<0.001	14	30.19	33	11.79	2.257	1.195	4.265	0.012
**Heart disease**	557	1,272.26	1,484	563.44	1.890	1.713	2.085	<0.001	14	31.98	32	12.15	2.320	1.229	4.385	<0.001
**CKD**	559	1,226.09	1,487	544.84	1.883	1.707	2.078	<0.001	14	30.71	32	11.72	2.309	1.222	4.363	<0.001
**Season**																
Spring	128	1,279.80	368	572.93	1.869	1.694	2.062	<0.001	4	39.99	9	14.01	2.516	1.332	4.755	<0.001
Summer	153	1,275.16	379	525.13	2.032	1.842	2.242	<0.001	1	8.33	9	12.47	0.589	0.312	1.113	0.413
Autumn	163	1,167.24	375	472.75	2.066	1.873	2.280	<0.001	3	21.48	8	10.09	1.878	0.994	3.549	0.058
Winter	117	1,117.57	370	573.81	1.630	1.477	1.798	<0.001	6	57.31	7	10.86	4.654	2.464	8.795	<0.001
**Urbanization level**																
1 (The highest)	178	1,242.98	489	554.34	1.877	1.701	2.070	<0.001	5	34.92	11	12.47	2.468	1.307	4.665	<0.001
2	269	1,286.41	684	559.40	1.925	1.744	2.123	<0.001	3	14.35	11	9.00	1.406	0.744	2.657	0.632
3	51	1,079.30	112	420.19	2.150	1.948	2.372	<0.001	1	21.16	4	15.01	1.243	0.658	2.349	0.481
4 (The lowest)	63	972.66	207	480.65	1.694	1.535	1.868	<0.001	5	77.20	7	16.25	4.187	2.217	7.912	<0.001
**Level of care**																
Hospital center	179	1,313.51	502	580.05	1.895	1.718	2.091	<0.001	3	22.01	5	5.78	3.359	1.779	6.348	<0.001
Regional hospital	233	1,172.03	540	494.49	1.984	1.798	2.188	<0.001	4	20.12	16	14.65	1.211	0.641	2.288	0.682
Local hospital	149	1,152.72	450	532.79	1.811	1.641	1.998	<0.001	7	54.15	12	14.21	3.360	1.779	6.350	<0.001

PYs = Person-years; Rate: per 10^5^ PYs; Adjusted HR = Adjusted Hazard ratio: Adjusted for the variables listed in Table 3.; CI = Confidence interval

Despite the other factors, patients with a history of PID had HRs for preterm labor ranging from 1.410 (P<0.001) to 2.772 (P<0.001), which were significantly different compared with the values of those without a history of PID. The same status was also noted in the population with ectopic pregnancies; despite the other factors, patients with a history of PID had HRs ranging from 1.753 (P<0.001) to 4.654 (P<0.001), which were significantly different compared with the values of those without a history of PID.

## Discussion

Pelvic inflammatory disease (PID) is an inflammatory condition of the female upper genital tract and includes a combination of endometritis, salpingitis, tubo-ovarian abscess, and pelvic peritonitis[34,35]. Approximately forty-one patients with acute pelvic inflammatory disease were evaluated for the coexistence of bacterial vaginosis. Due to the inflammatory process, tubal adhesion and intra-abdominal adhesion are observed after PID[36–41]. PID sometimes progresses to liver capsule inflammation and leads to the development of adhesions and Fitz-Hugh-Curtis syndrome [42–44]. For PID patients, long-term medical treatment, regular follow-up and good compliance are important. However, patients with PID can easily relapse, and it is difficult to achieve complete treatment; poor compliance is often noted in clinical practice [26,45]. The treatment of PID also places a substantial cost burden on the health care system. In developed countries, the annual incidence of PID in women 15 to 39 years of age is approximately 10 to 13 per 1,000 women, with a peak incidence of approximately 20 per 1,000 in women 20 to 24 years of age[46]. Moreover, complications of PID also place an extensive burden on the health care system. Medical costs of treatment have been estimated to be $166 million for chronic pelvic pain, $295 million for ectopic pregnancy, and $360 million for infertility associated with PID in the USA[47]. Preventative PID measures are thought to be more cost-effective [47].

By understanding the association of PID with preterm labor and ectopic pregnancy, assistance and intervention strategies for the clinical prevention of poor outcomes could be provided [19,20,30,48,49]. Some studies revealed that infection could be a precursor to preterm birth or to the premature rupture of membranes. Preexisting infection of the uterine cavity is a predisposing factor of premature membrane rupture, preterm delivery, and amnionitis[23,29,32]. However, there have been no large-scale studies on the relationship between previous PID and preterm labor in Taiwan or other Asian countries.

The role of salpingitis in the recurrence of ectopic pregnancy was studied in a historical cohort of 2,501 women who had undergone laparoscopic examination for acute salpingitis. The study concluded that salpingitis was a risk factor for first ectopic pregnancy[9]. However, there have been no large-scale studies of the relationship between previous PID and ectopic pregnancy in Taiwan or other Asian countries.

This nationwide, population-based study is a large-scale study that investigates the association of PID with ectopic pregnancy and preterm labor. We confirmed that PID is a significant risk factor for ectopic pregnancy and preterm labor. The PID population is at higher risk of ectopic pregnancy and preterm labor compared to the general population. Among PID patients, patients aged 12–19 years have a higher risk of developing ectopic pregnancy and preterm labor than other age groups. Some infectious diseases are pandemic diseases with obvious seasonal characteristics, and outbreaks occur periodically. The results showed that there was no significant difference between seasons. PID is not a pandemic disease.

In addition, patients with hypertension, heart disease, and CKD have an approximately 1.057 to 1.404 times higher risk of preterm labor. Therefore, hypertension, heart disease, and CKD are contributing factors to the development of preterm labor in patients with PID. People with poor health or hygiene problems may have more serious problems or consequences. Patients with hypertension, heart disease, and CKD have the potential for high-risk pregnancies with other complications. With the development of preeclampsia, eclampsia, general edema, or severe dyspnea complicated by heart disease and CKD, delivery is considered an important treatment, and preterm delivery may occur. From the analysis of related factors, we know that women of a young age are at the highest risk of PID. Sexual risk behavior is a critical problem in adolescents. Adolescents engaging in early and unsafe sexual activities represent a high-risk population for infection with human immunodeficiency virus, other sexually transmitted diseases, and unplanned pregnancy[50]. Adolescents who experience PID are highly likely to experience adverse reproductive health outcomes [51,52]. This outcome is an important issue in the prevention of PID[51–53]. The prevention of pelvic inflammatory disease, including comprehensive sex education, the promotion of condom use, and the provision of condoms, is a cornerstone in the prevention of sexually transmitted infection globally[54]. Our results showed that PID had a more prominent role in preterm labor and ectopic pregnancy than other factors; therefore, we consider PID to be a potential risk factor for preterm labor or ectopic pregnancy. Further study of PID should focus on improving disease detection, implementing cheap and effective treatments and, specifically, understanding the pathophysiology.

The strengths of our study include its population-based design, the use of well-established cohort data with a large sample size and the extended follow-up period used to identify PID as a risk factor for developing preterm labor and ectopic pregnancy. Nevertheless, there are still some limitations of this study. First, although the coding of the NHIRD has been validated for some diseases, no reports are available regarding the coding severity of PID. The infectious pathogens and microbiology involved in PID were also unable to be retrieved via the databank. At the same time, the effect on the severity of preterm labor or the site of ectopic pregnancy could not be analyzed. Second, the NHIRD registry was not able to provide detailed information regarding the laboratory results, health-related lifestyle or past history of the patients, such as smoking status, body mass index, gynecological history and family history, some of which can increase the risk of preterm or ectopic pregnancy. Third, the incidence of PID may be underestimated because patients without a hospital visit or a diagnosis of subclinical pelvic inflammatory disease[5] could not be identified from the Taiwan NHI data set. Fourth, the study was based on outpatient and inpatient data, which may not represent the general population (S2 Table).

## Conclusions

This study demonstrated that PID is a significant and independent risk factor for preterm labor and ectopic pregnancy. Compared with those without PID disease, patients with PID history had a 1.864 times (P<0.001) higher risk of developing preterm labor and a 2.121 times (P = 0.003) higher risk of developing ectopic pregnancy. The effect of disease progression on the development of preterm labor and/or ectopic pregnancy needs to be further elucidated in future studies. The results from this study indicate that clinical doctors need to perform a cautious assessment of PID patients with pregnancy problems.

## Supporting information

S1 Table Number of birth for events with and without PID. (DOCX)

S2 Table Factors of preterm labor / ectopic pregnancy subgroup by using Cox regression. (DOCX)
