# Supplementary material for: Association of pelvic inflammatory disease (PID) with ectopic pregnancy and preterm labor in Taiwan: A nationwide population-based retrospective cohort study
Source: PLoS One. 2019 Aug 13;14(8):e0219351. doi: 10.1371/journal.pone.0219351 (PMC6692029; doi:10.1371/journal.pone.0219351)
Supplement: S1 Table — (DOCX) [file pone.0219351.s001.docx]

**S1 Table. Number of birth for events with and without PID**

|  | **PID** | **Total** | | **With** | | **Without** | |
| --- | --- | --- | --- | --- | --- | --- | --- |
| **Events** | **Number of birth** | **n** | **%** | **n** | **%** | **n** | **%** |
| **Preterm labor** | **Overall** | 2,053 |  | 561 |  | 1,492 |  |
|  | **1** | 1,523 | 74.18 | 412 | 73.44 | 1,111 | 74.46 |
|  | **2** | 528 | 25.72 | 149 | 26.56 | 379 | 25.40 |
|  | **3** | 2 | 0.10 | 0 | 0.00 | 2 | 0.13 |
| **Ectopic pregnancy** | **Overall** | 47 |  | 14 |  | 33 |  |
|  | **1** | 34 | 72.34 | 10 | 71.43 | 24 | 72.73 |
|  | **2** | 12 | 25.53 | 3 | 21.43 | 9 | 27.27 |
|  | **3** | 1 | 2.13 | 1 | 7.14 | 0 | 0.00 |
