# Supplementary material for: Association of pelvic inflammatory disease (PID) with ectopic pregnancy and preterm labor in Taiwan: A nationwide population-based retrospective cohort study
Source: PLoS One. 2019 Aug 13;14(8):e0219351. doi: 10.1371/journal.pone.0219351 (PMC6692029; doi:10.1371/journal.pone.0219351)
Supplement: S2 Table — (DOCX) [file pone.0219351.s002.docx]

**S2 Table. Factors of preterm labor / ectopic pregnancy subgroup by using Cox regression**

|  | **Year** | **2000-2010** | | | |  | **2000-2013** | | | |
| --- | --- | --- | --- | --- | --- | --- | --- | --- | --- | --- |
|  | **PID (With vs. Without)** | **Adjusted HR** | **95% CI** | **95% CI** | **P** |  | **Adjusted HR** | **95% CI** | **95% CI** | **P** |
| **OPD / ER / IPD** | **Overall** | 1.875 | 1.711 | 2.098 | <0.001 |  | 1.832 | 1.701 | 2.088 | <0.001 |
|  | **Preterm labor** | 1.864 | 1.482 | 2.062 | <0.001 |  | 1.855 | 1.445 | 1.986 | <0.001 |
|  | **Ectopic pregnancy** | 2.121 | 1.803 | 3.776 | 0.003 |  | 2.097 | 1.798 | 3.512 | <0.001 |
| **OPD / ER** | **Overall** | 1.862 | 1.605 | 2.061 | <0.001 |  | 1.795 | 1.582 | 1.999 | <0.001 |
|  | **Preterm labor** | 1.833 | 1.334 | 2.033 | <0.001 |  | 1.731 | 1.312 | 1.842 | <0.001 |
|  | **Ectopic pregnancy** | 2.010 | 1.698 | 3.567 | 0.005 |  | 1.902 | 1.599 | 3.311 | <0.001 |
| **IPD** | **Overall** | 1.976 | 1.802 | 2.151 | <0.001 |  | 1.964 | 1.772 | 2.103 | <0.001 |
|  | **Preterm labor** | 1.897 | 1.563 | 2.095 | <0.001 |  | 1.885 | 1.458 | 1.997 | <0.001 |
|  | **Ectopic pregnancy** | 2.206 | 1.816 | 3.810 | <0.001 |  | 2.130 | 1.811 | 3.401 | <0.001 |
